# Mr. Clark's Patent Method of Inserting Pivot Teeth

**Published:** 1849-07

**Authors:** 


*Mr. Clark's Patent Method of Inserting Pivot Teeth.*—The Dental Recorder, of March, 1849, contains a description of a method, patented by Mr. Clark of New York, of inserting pivot teeth. It consists in the introduction of a hollow cylinder into the opening usually made for the reception of a wood pivot. This is held in its place by a screw passed through the bottom into the canal of the root; on the side of the cylinder is a catch for the pivot. That the principle is original with Mr. Clark, we do not for a moment doubt; and while we would not wish to detract in the least from his merit as an ingenious and skilful practitioner, candor compels us to state, that the invention, so far as its application to the insertion of artificial teeth is concerned, is due to Maggiolo, author of a small treatise on the Art of the Dentist, published in Nancy, January, 1807. In chapters 9 and 10 of this work, is a description of the *catch* method of confining the pivot of an artificial crown in the root of a natural tooth, previously lined with a gold tube; and in plate first of the same work, the tube, with the catch, pivot and artificial crown are represented. This method, too, is alluded to by Delabarre, in the second volume of his treatise on Mechanical Dentistry, published in Paris, 1820, page 369, and by Desirabode, as well as other French writers.

Now, although Mr. Clark was not the first to apply the principle here alluded to, to the insertion of pivot teeth, and consequently not entitled to the exclusive privilege of using it, his letters patent to the contrary notwithstanding, we think it likely, so far as we can understand the description as given by our friend, the editor of the Dental Recorder, that his pivot and its connection to the crown of the tooth, is more perfect in its construction than the one employed by Maggiolo.

But the time necessarily occupied in the construction, and accuracy required in the perfect adaptation of the different parts to each other, has hitherto prevented, and will continue to prevent, the frequent application of pivot teeth upon this principle. In France, where it originated, it has been long since abandoned, not so much on account of the time required for the insertion of a tooth in this way, but because the principle was not found to be as good as some others which have been adopted. The introduction of a hollow screw into the root, large enough for the reception of a sufficiently large gold pivot, a method that has been long practiced, possesses all the advantages, not only as it regards the protection of the walls of the root against the action of the secretions of the mouth, but for the secure retention of the tooth, as does the clacking pivot of Maggiolo and Mr. Clark. The procedure necessary for the insertion of a tooth in this way, we described, during a conversation with several professional friends, in the office of Dr. Jahial Parmly, of New York, some two or three years ago, and to the class of the Baltimore College of Dental Surgery, with the necessary illustrations, during every session for the last eight or nine years. It has also been practiced, under our own direction, by several of the students. Now, while this is a very simple method, we regard it as the best that has ever been adopted in the application of an artificial crown to the root of a natural tooth.—*Balt. Ed.*

